# Evaluation of important phenotypic parameters of tea plantations using multi-source remote sensing data

**DOI:** 10.3389/fpls.2022.898962

**Published:** 2022-07-22

**Authors:** He Li, Yu Wang, Kai Fan, Yilin Mao, Yaozong Shen, Zhaotang Ding

**Affiliations:** ^1^Tea Research Institute, Qingdao Agricultural University, Qingdao, China; ^2^Tea Research Institute, Shandong Academy of Agricultural Sciences, Jinan, China

**Keywords:** UAV, multispectral, LiDAR, RGB, thermal, tilt photography, tea plants phenotype

## Abstract

Tea height, leaf area index, canopy water content, leaf chlorophyll, and nitrogen concentrations are important phenotypic parameters to reflect the status of tea growth and guide the management of tea plantation. UAV multi-source remote sensing is an emerging technology, which can obtain more abundant multi-source information and enhance dynamic monitoring ability of crops. To monitor the phenotypic parameters of tea canopy more efficiently, we first deploy UAVs equipped with multispectral, thermal infrared, RGB, LiDAR, and tilt photography sensors to acquire phenotypic remote sensing data of tea canopy, and then, we utilize four machine learning algorithms to model the single-source and multi-source data, respectively. The results show that, on the one hand, using multi-source data sets to evaluate H, LAI, W, and LCC can greatly improve the accuracy and robustness of the model. LiDAR + TC data sets are suggested for assessing H, and the SVM model delivers the best estimation (Rp^2^ = 0.82 and RMSEP = 0.078). LiDAR + TC + MS data sets are suggested for LAI assessment, and the SVM model delivers the best estimation (Rp^2^ = 0.90 and RMSEP = 0.40). RGB + TM data sets are recommended for evaluating W, and the SVM model delivers the best estimation (Rp^2^ = 0.62 and RMSEP = 1.80). The MS +RGB data set is suggested for studying LCC, and the RF model offers the best estimation (Rp^2^ = 0.87 and RMSEP = 1.80). On the other hand, using single-source data sets to evaluate LNC can greatly improve the accuracy and robustness of the model. MS data set is suggested for assessing LNC, and the RF model delivers the best estimation (Rp^2^ = 0.65 and RMSEP = 0.85). The work revealed an effective technique for obtaining high-throughput tea crown phenotypic information and the best model for the joint analysis of diverse phenotypes, and it has significant importance as a guiding principle for the future use of artificial intelligence in the management of tea plantations.

## Introduction

Tea [*Camellia sinensis (L.) O. Kuntze*] is an evergreen cash crop, which is widely cultivated all over the world. Phenotypic parameters, such as height (H), leaf area index (LAI), leaf water content (W), leaf chlorophyll, and nitrogen concentration (LCC and LNC), are important indicators to estimate the growth and development of tea plants. Using these parameters on large scale can effectively guide the daily management of tea plantations in field. However, the conventional measurement methods of these parameters have some problems, such as time-consuming and labor-intensive, low efficiency, high cost, and poor accuracy, which seriously restrict growth, development, and smart yield of tea on larger scale. Therefore, it is important to find better, faster, and smarter way of phenotyping methods for more accurate results.

In recent years, with the rise of new remote sensing tools around the world, UAV system has gradually become an important means to obtain the phenotypic information of field crops with the advantages of flexibility, adaptability to complex farmland environment, high efficiency, and low cost (Herwitz et al., 2004). At the present, UAV sensors mostly comprise optical sensors, thermal sensors, and three-dimensional reconstruction sensors.

As we know that, optical sensors could obtain spectral reflectance and texture information of vegetation (Kalaitzidis et al., 2008; Chianucci et al., 2016), and optical information had been successfully applied to crop phenotype analysis. For example, the density of maize plants was estimated using UAV RGB data (Štambuk et al., 2021). The vegetation index of soybean canopy was obtained by UAV RGB image, and the canopy volume model was constructed to estimate soybean biomass (Maimaitijiang et al., 2019). The hyperspectral image of UAV and the lodging characteristics of rice were used to establish the rice yield detection model (Wang et al., 2021). The UAV multispectral data were used to estimate the biochemical components of tea canopy leaves (Luo et al., 2021). However, the optical sensors that were utilized to monitor the dense field crops suffered from the challenge of progressive saturation, which caused it harder to obtain the structural parameters and canopy temperature of the crops.

The thermal sensor could be able to obtain the canopy temperature of field crops. The obtained temperature information had a high correlation with plant water content in field. Therefore, thermal sensors were mostly used to monitor the temporal and spatial changes in crop water content and evaluate the drought degree of crops (Abdelhakim et al., 2021). Some researchers also use thermal infrared data to evaluate plant leaf area index and chlorophyll content (Lin et al., 2021). Infrared imaging is mainly used to monitor the water content of plants. The monitoring of other phenotypic parameters needs to be further explored.

To get accurate information on crop canopy structure, the three-dimensional reconstruction sensor is the fundamental sensor. The three-dimensional reconstruction sensor is the main sensor to obtain the crop canopy structure information. It generates point cloud data through the structure from motion (SFM) to establish a three-dimensional (3D) model (Brook et al., 2021). There are two main methods to build 3D models. One is to obtain omnidirectional images, *via* multi-angle oblique photography technique, and then splice the omnidirectional images to establish a three-dimensional model. The other is to launch the laser beam through the LiDAR sensor and then locate the laser beam hitting the spot of the object to establish the three-dimensional model (Perez and Costes, 2018). These two methods have made good progress in agricultural application: for instance, combining oblique and vertical photography technologies from a UAV to create a 3D model to estimate the plant height and leaf area of maize growing in a field (Ying et al., 2020), and using 3D rotating LiDAR sensor to establish a three-dimensional model to estimate the canopy density of perennial horticultural crops (Lowe et al., 2021). In contrast, the tilt photography sensor has low cost and is suitable for large-scale popularization in the field. However, this is a passive technology with less density and information than the point cloud generated by LiDAR sensors (Luo et al., 2019).

In recent years, the continuous development of computer hardware has facilitated the progress of machine learning, which has become an active research area in agricultural quantitative remote sensing (Liu et al., 2021). For example, Luo et al. (2021) used the UAV equipped with multispectral sensors to obtain the spectral data of tea canopy leaves and used support vector regression (SVR) and partial least square regression (PLSR) to estimate the nitrogen content, polyphenols, and caffeine. Liu et al. (2021) used the UAV equipped with multispectral, RGB and thermal infrared to obtain the multi-source data of corn canopy and established regression models using RNN, PLS, RF, and SVM to evaluate the LAI.

Single-source remote sensing has made good progress in monitoring various parameters of crops, but it has limitations. Because the information collected by different sensors is different, if only relying on a single sensor to monitor crops, some data will be lost, which will affect the accuracy of the model. Moreover, in the study of maize and soybean phenotypes, it has been proved that the accuracy of modeling with multi-source data is higher than that with single-source data, as shown in Table 1. Therefore, it is of great significance to study the multi-source remote sensing monitoring of tea plant growth indicators.

**Table 1:** Results of estimating relevant indexes of field crops using multi-source remote sensing data.

**Sensor type**	**Phenotypic**	**Crop**	**Algorithm**	**Accuracy**	**References**
RGB	LAI	Maize	DCNN	R^2^ = 0.82	Liu et al., 2021
			RF	R^2^ = 0.71	
MS	LAI	Maize	DCNN	R^2^ = 0.7	
			RF	R^2^ = 0.68	
TM	LAI	Maize	DCNN	R^2^ = 0.51	
			RF	R^2^ = 0.54	
RGB+MS+TM	LAI	Maize	DCNN	R^2^ = 0.89	
			RF	R^2^ = 0.76	
LiDAR	H	Maize	RF	R^2^ = 0.76	Yue et al., 2018
	AGB		SVM	R^2^ = 0.74	
RGB	H	Maize	RF	R^2^ = 0.69	
	AGB		SVM	R^2^ = 0.64	
HS	H	Maize	RF	R^2^ = 0.56	
	AGB		SVM	R^2^ = 0.54	
LiDAR+RGB+MS	H	Maize	RF	R^2^ = 0.82	
	AGB		SVM	R^2^ = 0.8	
MS	AGB	Soybean	SVM	R^2^ = 0.52	Maimaitijiang et al., 2020
RGB	AGB	Soybean	SVM	R^2^ = 0.42	
TM	AGB	Soybean	SVM	R^2^ = 0.26	
MS+RGB+MS	AGB	Soybean	SVM	R^2^ = 0.67	

In this research work, we smartly use multi-source remote sensing data, including RGB images, multispectral images, TM images, LiDAR images, and TC images, collected from a UAV crop high-throughput phenotyping platform, to develop a multimodal data processing framework to estimate the H, LAI, W, LCC, and LNC of tea plants in field. The proposed framework is mainly based on four key machine learning methods: back propagation (BP), support vector machine (SVM), random forest (RF), and partial least squares (PLS). This work makes several contributions: (1) It proposes a framework for processing fused, multi-source remote sensing data, which produces multimodal data sets to improve estimates of the H, LAI, W, LCC, and LNC; (2) comparing the robustness and adaptability of multi-mode data fusion and single-source data evaluation models to estimate H, LAI, W, LCC, and LNC of tea plant and found an optimal estimation model for different phenotypes; (3) the tea phenotypic model constructed by RF and SVM algorithm has the highest accuracy and robustness.

## Materials and methods

### Study area

**Figure 1:**
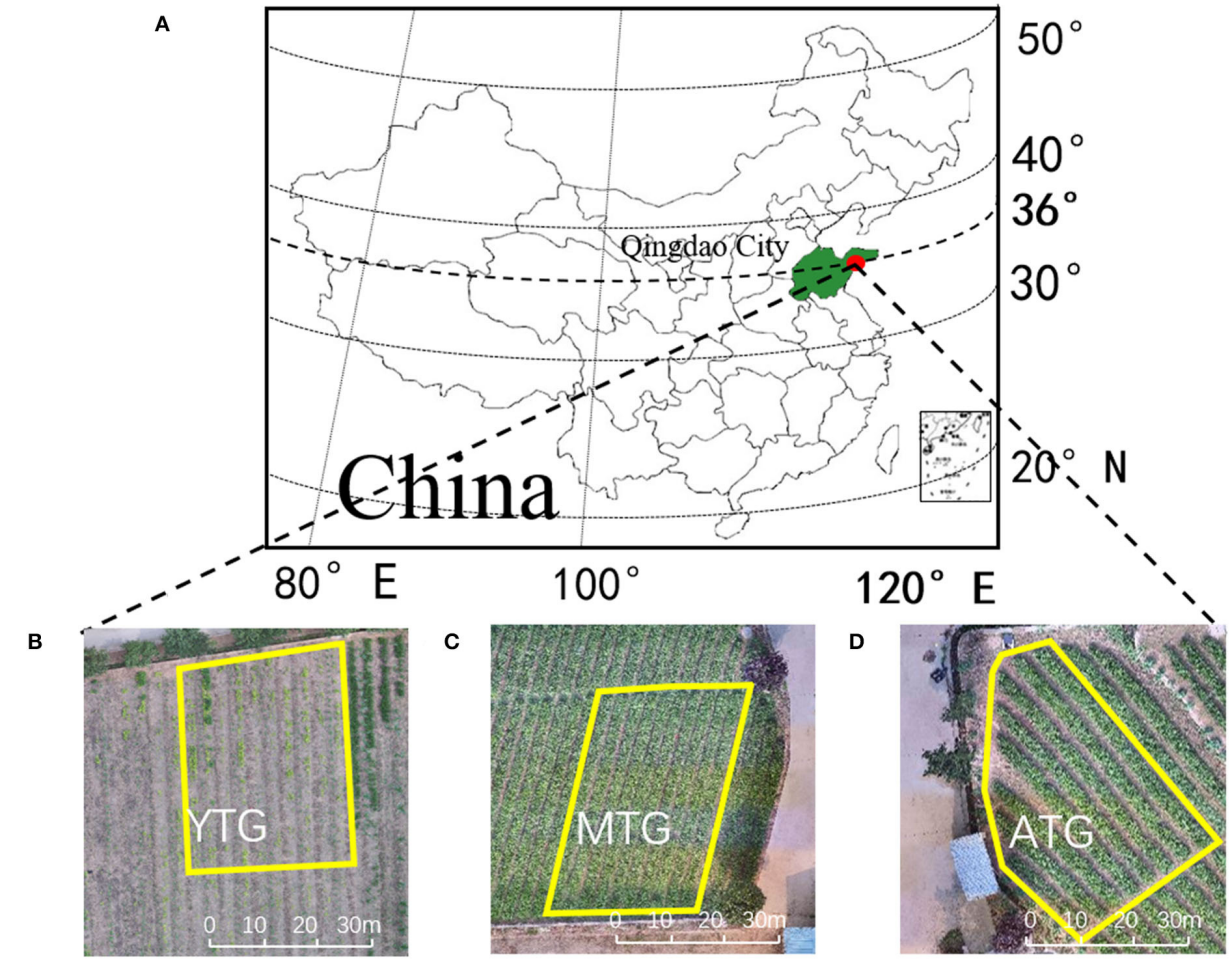
**(A)** Geographical location of the study area (Qingdao); **(B)** young tea garden (YTG); **(C)** mature tea garden (MTG); **(D)** aging tea garden (ATG).

The study areas were located in Bi Hai Lan Tian Tea plantations (120.61°E, 36.27°N, Figure 1), Laoshan District, Qingdao City, Shandong Province. It covers an area of about 65 hectares, with an average altitude of 55 meters. The soil texture is sandy, the unit weight is 1.45 g/cm^3^, the organic matter is 1.63%, and the pH value is 6.0. The annual average precipitation is 719.2 mm, the annual average sunshine hours are 2,392 h, and the annual average temperature is 13.5°C (the annual maximum/minimum temperature is 39.6/−19.6°C), which is suitable for the growth and development of tea plants. The experimental areas were divided into three tea plantations at different growth stages, the age of tea plants in young tea garden (YTG) is 4 years old, mature tea garden (MTG) is 10 years old, and aging tea garden (ATG) is 22 years old. The location of the test area is shown in Figure 1.

The field experiment was conducted in November 2020. The three tea plantations were divided into experimental units. YTG is divided into 70 test subunits, each of which is 3 m^2^; MTG is divided into 50 test subunits, each with 4 m^2^; ATG is divided into 60 test subunits, each of which is 3 m^2^. Three tea plantations were watered and fertilized. YTG watering is 45 mm, MTG watering is 120 mm, and ATG watering is 120 mm. Drip irrigation is adopted. The fertilizer is organic fertilizer. Mechanical trenching is adopted for fertilization. YTG is applied with 75 kg hm^−2^, MTG is applied with 112 kg hm^−2^, and ATG is applied with 85 kg hm^−2^.

### Data collection

#### Field data collection

**Figure 2:**
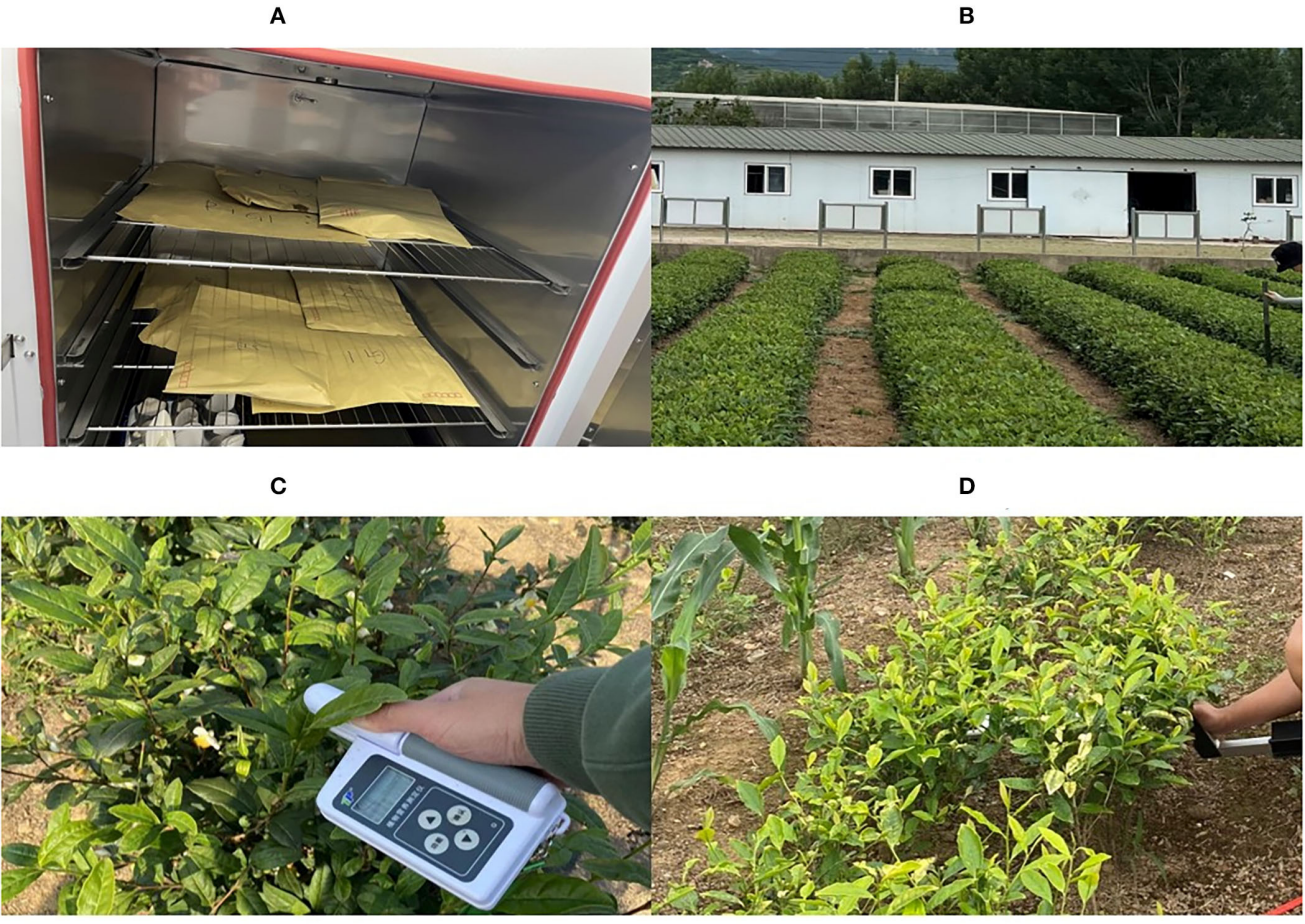
**(A)** Determination of the W; **(B)** determination of the H; **(C)** determination of the LCC and LCN; **(D)** determination of the LAI.

Field data were collected on July 1, 2021, and five tea plant phenotypic parameters were measured in this study, including LAI (m^2^ m^−2^), H (m), W (%), LCC (SPAD value), and LNC (mg g^−1^) (Table 2). All five parameters were measured between July 1 and July 2, 2021 (Figure 2). All tea plants in the tea gardens were pruned, and the fresh shoots were picked before phenotypic parameters were measured and flight missions were performed. As a result, our measurements of leaf values are based on mature leaves. To verify the typicality of the collected samples and minimize measurement error, we randomly measure the leaves of multiple tea plants in the test unit and then use the average as the final input data.

**Table 2:** Descriptive statistics were used to analyze the phenotypic parameters of tea plantations.

**Tea parameters**	**Number**	**Min**	**Mean**	**Max**	**SD**
LAI (m^2^ m^−2^)	Field all = 180	0.53	2.4	5.10	1.31
	Field YTG = 70	0.53	1.14	2.07	0.35
	Field MTG = 60	3.23	4.13	5.10	0.42
	Field ATG = 50	1.1	2.41	4.03	0.71
H (m)	Field all = 180	0.21	0.36	0.52	0.079
	Field YTG = 70	0.21	0.34	0.46	0.054
	Field MTG = 60	0.40	0.47	0.52	0.025
	Field ATG = 50	0.22	0.31	0.38	0.036
W (%)	Field all = 180	61	68.54	76	3.91
	Field YTG = 70	68	72.40	76	1.52
	Field MTG = 60	61	66.03	73	2.64
	Field ATG = 50	62	66.11	75	2.91
LCC (SPAD)	Field all = 180	61.3	69.23	76.5	4.22
	Field YTG = 70	61.3	64.57	75.2	2.09
	Field MTG = 60	65.4	71.76	75.3	1.97
	Field ATG = 50	68.1	72.56	76.5	1.75
N (mg g^−1^)	Field all = 180	17.1	20.88	26.4	1.59
	Field YTG = 70	19.7	22.07	26.4	1.31
	Field MTG = 60	17.1	20.10	23.8	1.46
	Field ATG = 50	17.4	20.14	23.6	1.10

The LAI was measured by the plant canopy Digital Image Analyzer CI-110 (CID USA). The final result is the average value of three measurements in each test area (Brand and Zonta, 2016). The H was measured by hand using a ruler, and the final result is the average of six measurements taken in each test area. The W was measured by oven. Ten mature leaves from each test area were taken and dried to constant weight in an oven at 90°C to constant weight. The LCC and LNC were measured by plant Nutrition analyzer (Tuopu Zhejiang, China), carefully avoiding the leaf veins during whole measurement for accuracy of results. The final average data were calculated according to the prescribed formula of W as follows:


(1) $$W\;=\;\frac{m1-m2}{m2}\times 100\%$$

where m1(g) is the total weight of the blade, and m2 (g) is the weight after drying.

#### UAV multi-sensor data acquisition

**Figure 3:**
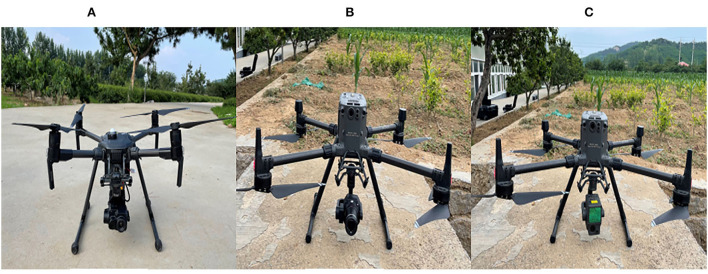
**(A)** M200 V2 carries with MS600 and Meditation XT2; **(B)** the M300 RTK carries Meditation P1; **(C)** the M300 RTK carries with Meditation L1.

To ensure flight quality and safety, we choose sunny weather and low wind speed conditions to perform the flight mission. On July 1, 2021, three UAVs were equipped with four sensors to perform flight tasks (Figure 3). The DJ M300 RTK (DJI, Inc., Shenzhen, China) was equipped with Meditation L1 (DJI, Inc., Shenzhen, China) and Meditation P1 (DJI, Inc., Shenzhen, China), respectively. DJ M200 V2 (DJI, Inc., Shenzhen, China) was equipped with MS600 (Yusense, Inc., Qingdao, China) and Meditation XT2 (DJI, Inc., Shenzhen, China). The specific information about the UAV system and its flight mission are shown in Table 3.

**Table 3:** Specific information on UAV systems and their flight missions.

**UAV platforms**	**Sensors**	**Flight height (m)**	**Flying speed (m s** ^−1^ **)**	**Overlap** (**%**)	**Accuracy (cm pixel^−1^)**
M300 RTK	Meditation L1	50	6	70 (front) 80 (side)	0.8
M300 RTK	Meditation P1	50	6	70 (front) 80 (side)	0.7
M200 V2	MS600	15	2	55 (front) 75 (side)	1.2
M200 V2	Meditation XT2	15	2	55 (front) 75 (side)	1.0

### Further processing of UAV remote sensing data

**Figure 4:**
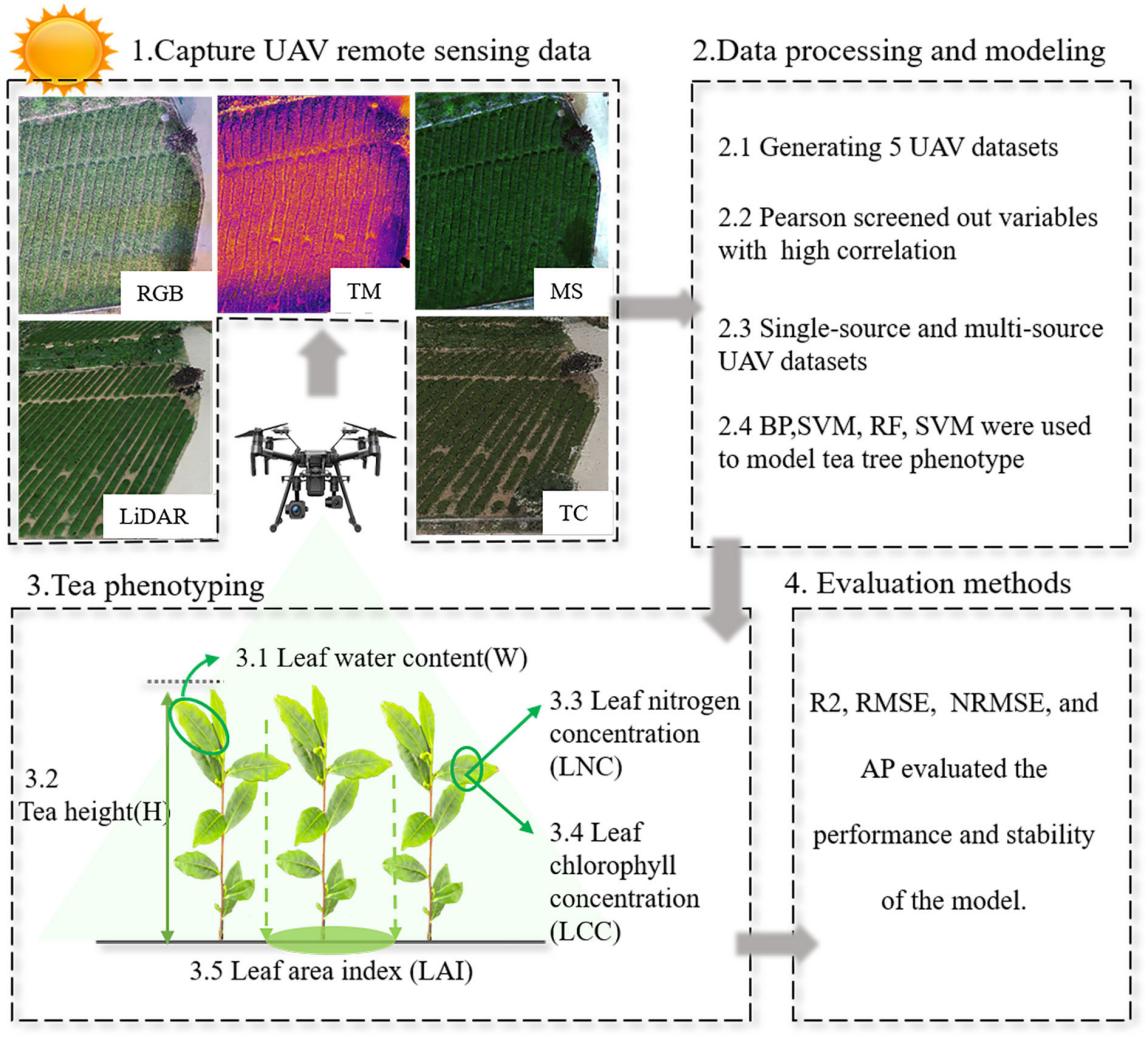
General framework for evaluating tea phenotype based on multi-source remote sensing data.

Figure 4 shows the overall framework for evaluating tea phenotype based on multi-source remote sensing data. First, 65 variables are extracted from LiDAR, TC, MS, RGB, and TM images. Second, the variables were screened by Pearson's correlation analysis. Then, using four machine learning methods, five tea phenotypic data are used to model the selected variables. R^2^, RMSE, and NRMSE are used to evaluate the quality of the model. To eliminate the influence of flight altitude on the data set, we extract the marked coordinate points from the LiDAR image and input the marked coordinate points in other images during the splicing process.

a) The LiDAR data collected by Meditation L1 were used to establish point cloud model by DJI SmartMap software (DJI, Inc., Shenzhen, China). The processing process includes screening the high-density point cloud, output coordinate system location CGRS93, point cloud accuracy optimization, and reconstruction.
b) The TC data collected by Meditation P1 were used to establish point cloud model by DJI SmartMap software. The processing process includes selecting high-definition images, oblique and orthographic shooting scenes, and reshaping.
c) The MS data collected by MS600 were spliced by Yusense Map V1.0 (Yusense, Inc., Qingdao, China). The processing process includes generating the registration parameters for image registration, inputting white board reflectivity radiometric calibration, and splicing multispectral images (Luo et al., 2021).
d) The TM and RGB data collected by Meditation XT2 were spliced by Yusense Map V1.0. The processing process includes data import, camera parameter generation, image splicing, and temperature calibration.
e) For LiDAR and OC data, Alandur Platform Free software (ALD. Inc., Chengdu, China) was used to cut plots and extract variables. For MS and RGB data, ENVI 5.2 software was used for plot clipping, band, and texture extraction. For TM data, FLIR Tools (Teledyne FLIR, USA) software was used for cropping of plots and extraction of temperature information. For MS and RGB data, ENVI 5.2 software was used for plot clipping, band, and texture extraction. Python 3.7 and MATLAB 2020 were used for further processing and analysis of remote sensing data.

### The extraction of UAV remote sensing information

To clearly display the remote sensing indicators used in this article, we classify and rank the variables extracted from LiDAR, TC, MS, RGB, and TM data, as shown in Table 4.

**Table 4:** Definitions of the features extracted from different sensors and imagery.

**Sensor**	**Fentures**	**Formula**	**References**
LiDAR	Point cloud density	L.PCD = NOPC	Su et al., 2016
	Laser penetration index	L.LPI = NOGPC/NOGPC+NOCPC	
	Porosity	L.Fgap = NOGPCE/TNOPCE	
	Height mean	Mean height of tree crown	
	Height maximum	Maximum height of tree crown	
	Height percentile (5, 15, 25, 35, 45, 55, 65, 75, 85, 95%)	Percentile height of echo return point	
TC	Point cloud density	P.PCD = NOPC	Su et al., 2016; Ying et al., 2020
	Porosity	P.Fgap = NOGPE/TNOPE	
	Height mean	Mean height of tree crown	
	Height maximum	Maximum height of tree crown	
	Height percentile (5, 15, 25, 35, 45, 55, 65, 75, 85, 95%)	Percentile height of echo return point	
MS	450, 555, 660, 720, 750, 840 nm	The raw value of each band	
	Normalized difference vegetation index	NDVI = (NIR–R)/(NIR+R)	Peñuelas et al., 1997
	Ratio vegetation index	RVI = NIR/R	Jordan, 1969
	Difference vegetation index	DVI = NIR–R	Richardson and Wiegand, 1977
	Enhanced vegetation index	EVI = 2.5(NIR–R)/(NIR+6R−7.5B+1)	Hui and Huete, 1995
	Renormalized difference vegetation index	RDVI = (NIR–R)/($\sqrt{NIR+R}\;$)	Roujean and Breon, 1995
	Triangular vegetation index	TVI = 60(NIR–G) – 100(R–G)	Broge and Leblanc, 2001
	Soil-adjusted vegetation index	SAVI = 1.5(NIR–R)/(NIR+R+0.5)	Huete, 1988
	Nonlinear vegetation index	NIR = (NIR^2^-R)/(NIR^2^+R)	Goel and Qin, 1994
	Red-edge chlorophyll index	RECI = NIR/R−1	Gitelson et al., 2006
	Modified nonlinear vegetation index	MNLI = 1.5 (NIR^2^-R)/(NIR^2^+R+0.5)	Peng et al., 2003
	Optimization of soil-adjusted vegetation index	OSAVI = 1.16(NIR–R)/(NIR+R+0.16)	Rondeaux et al., 1996
	Green normalized difference vegetation index	GNDVI = (NIR–G)/(NIR +G)	Gitelson et al., 1996
	Red-edge NDVI	RENDVI = (R750–R720)/(R750+R710)	Gitelson and Merzlyak, 1996
RGB	Gray-level co-occurrence matrix (GLCM)	ME, VA, HO, CO, DI, EN, SE, CO	Haralick et al., 1973
TM	Temperature maximum	TMAX	Zhu et al., 2021
	Temperature minimum	TMIN	
	Temperature mean	TI/I	

*NP, number of point clouds; NOGPC, number of ground point clouds; NOCPC, number of canopy point clouds; NOGPCE, number of ground point clouds echoes; TNOPCE, total number of point cloud echoes*.

#### Extraction of LiDAR information

The point cloud model of LiDAR data was further processed by Alandur Platform Free software. The processing process includes denoising, filtering, normalization, and generating DSM model and DEM models. DSM model subtracts DEM model to further generate canopy height model (CHM). In this way, five variables can be extracted: point cloud density (PCD), laser penetration index (LPI), porosity (Fgap), height mean (Hmean), and height maximum (Hmax). The height information was related to the Z coordinate system of point cloud data. Therefore, the Z coordinate system of the point cloud model was rearranged by Python 3.8 to obtain a total of 10 variables of height percentile, namely H5th, H15th, H25th, H35th, H45th, H55th, H65th, H75th, H85th, and H95th. Therefore, the LiDAR data set contains 5 + 10 = 15 variables.

#### Extraction of TC information

The extraction of TC information was basically the same as that of LiDAR information, but there were no LPI variables. Thus, the TC data set contained 4 + 10 = 14 variables.

#### Extraction of MS information

MS data were extracted into six original bands through ENVI 5.2, including 450, 555, 660, 720, 750, and 840 nm. In addition, we applied 13 vegetation indices commonly used in previous studies. Therefore, the MS data set contains 6 + 13 = 19 variables.

#### Extraction of RGB information

Because MS data provide spectral information, we use high-resolution RGB data to extract texture information. The texture information was extracted from the gray-level co-occurrence matrix (GLCM) of green, red, and blue bands by ENVI 5.2 software, and the processing window is 3 lines ×3 columns. GLCM texture includes eight indexes: mean, variance, homogeneity, contrast, dissimilarity, entropy, second moment, and correlation. Therefore, the RGB data set includes 3 ×8 = 24 variables.

#### Extraction of TM information

For thermal sensors, temperature is the most important information. Therefore, we use FLIR Tools software to extract three temperature variables from TM data, namely temperature maximum (Tmax), temperature minimum (Tmin), and temperature mean (Tmean).

### Data modeling and validation

In this study, BP, SVM, RF, and PLS neural networks were used to analyze the data and establish the model. BP neural network had the ability of data integration and analysis, which could be used to analyze the nonlinear relationship between parameters affecting phenotypes (Liu et al., 2016). SVM had unique advantages in solving small sample, nonlinear, and high-dimensional pattern recognition problems, and its network structure is more complex, with strong generalization and prediction ability (Qin and He, 2005). PLS combined the advantages of principal component analysis, canonical correlation analysis, and multiple linear regression analysis and can handle the problem of multicollinearity between feature attributes (Lin et al., 2016). RF can balance errors for unbalanced data sets and has fast training speed, and it was easy to make a parallelization method (Iverson et al., 2008).

The variables of multi-source data sets and single-source data sets were screened by Pearson's correlation analysis, and the variables with high correlations were selected to be input into the four networks. To further expand the number of samples and ensure the accuracy of the algorithm, this study uses the method of 10-fold cross-validation to divide the data set into 10 parts, of which nine parts were used as the training set and one part was used as the test set, repeated 100 times, and finally calculate the average value of the results. The performance of the model was evaluated by determining R Square (R^2^), root mean square error (RMSE), and normalized root mean square error (NRMSE). The larger R^2^, the smaller RMSE and NRMSE, indicating the better performance of the model. The stability of the data set to different models was evaluated by average precision (AP). R^2^, RMSE, NRMSE, and AP were as follows:


(2) $$R^{2}\;=\;\frac{\sum_{i\;=\;1}^{n}{\left(x_{i}-\bar{x}\right)}^{2}\times {\left(y_{i}-\bar{y}\right)}^{2}}{\sum_{i\;=\;1}^{n}{\left(x_{i}-\bar{x}\right)}^{2}\times \sum_{i\;=\;1}^{n}{\left(y_{i}-\bar{y}\right)}^{2}}$$

(3) $$\;RMSE\;=\;\sqrt{\frac{\sum_{i\;=\;1}^{n}{\left(y_{i}-x_{i}\right)}^{2}}{n}\;}$$

(4) $$NRMSE\;=\;\frac{RMSE}{\bar{x}}\;$$

(5) $$AP\;=\;\frac{\sum_{i\;=\;1}^{n}R_{i}}{n}$$

## Results and analysis

### Phenotypic analysis of tea crowns at different growth stages

**Figure 5:**
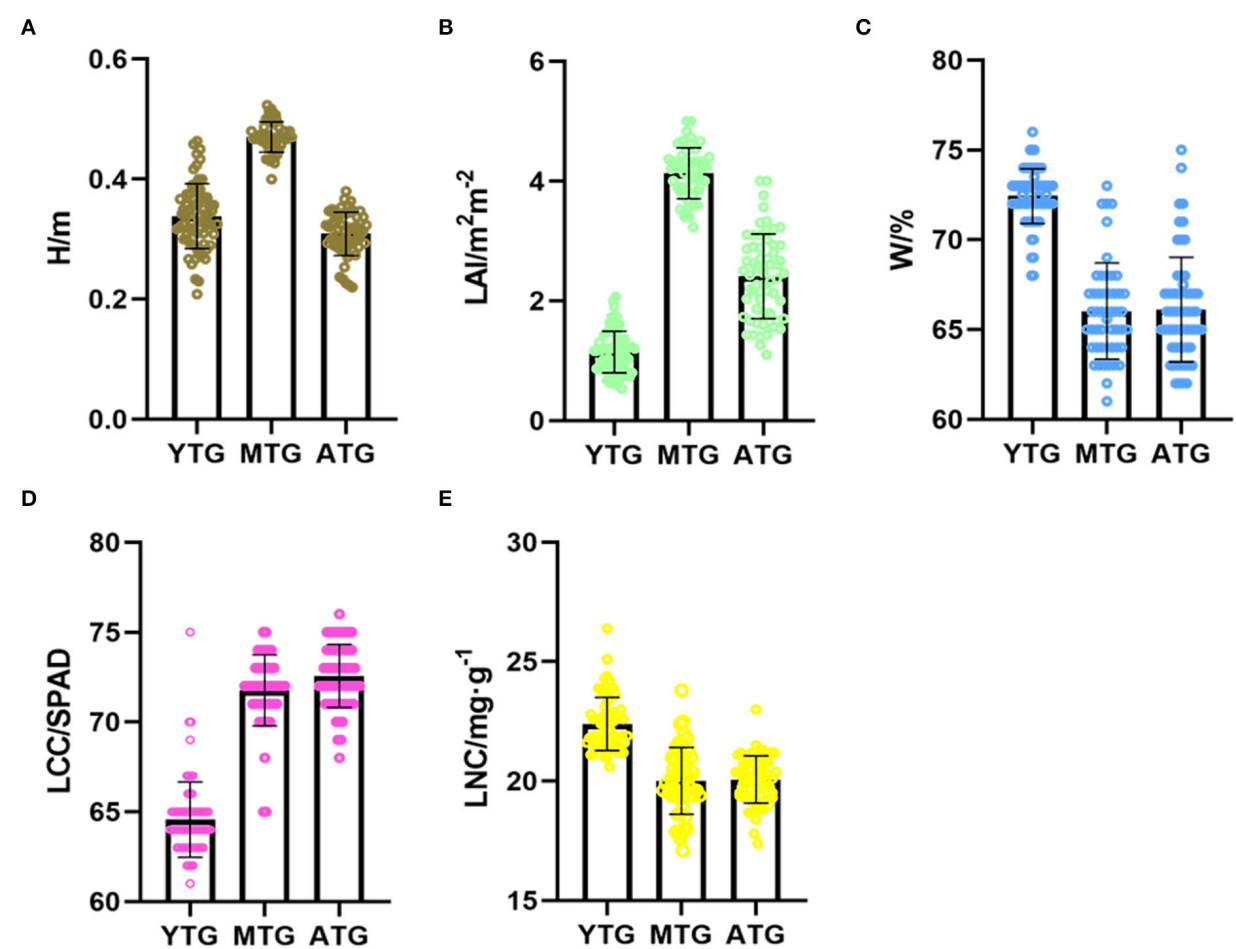
Phenotypes of tea crowns at different growth stages. **(A)** H; **(B)** LAI; **(C)** W; **(D)** LCC; **(E)** LNC.

To obtain the phenotypic information of tea crown, LAI, H, W, LCC, and LNC were measured by artificial method (Figure 5). The H of MTG is about 0.5 m, and the LAI is about 5 m^2^m^−2^, which is the largest among the three tea gardens, indicating that the canopy of MTG is the densest. The LAI of YTG is about 0.1 m^2^m^−2^, which is the smallest of the three tea gardens, indicating that the tea plant is in the growth stage. For W, the water content of tea leaves in YTG is the largest, which is 73%, and the W of MTG and ATG is lower. For LCC, the chlorophyll of ATG tea leaves is the largest, and the SPAD value is about 73. The chlorophyll of YTG tea leaves is the smallest, and the SPAD value is about 65. For LNC, the average LNC of MTG and ATG is about 20 mg g^−1^, which indicates that tea plants are seriously deficient in nitrogen.

### Contribution of single-source remote sensing data to estimation of tea crown phenotypic

#### Screening of single-source UAV remote sensing data

**Figure 6:**
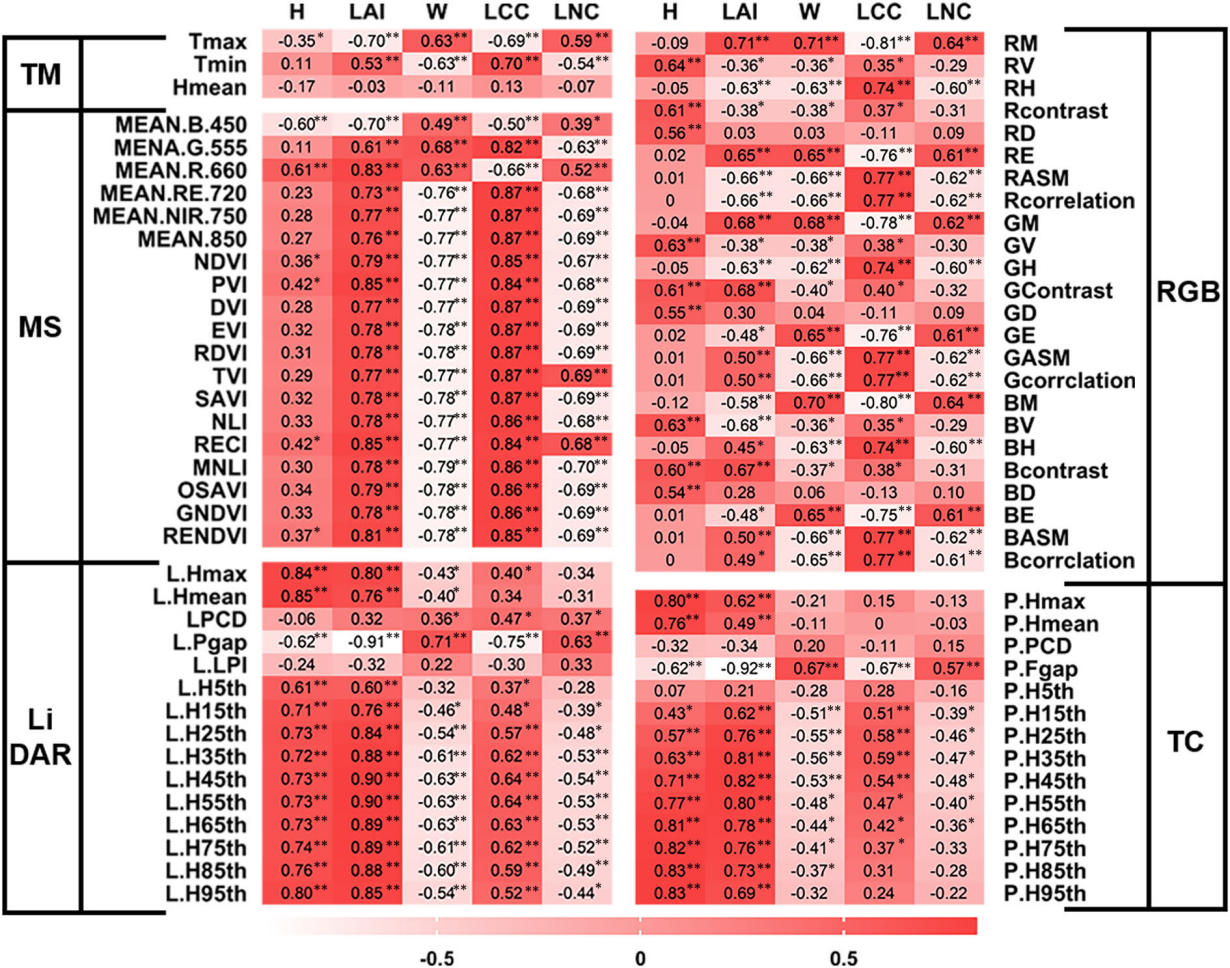
Selected single-source remote sensing variables with high correlation. * and ** represent the significance levels of *P* < 0.05 and *P* < 0.01, respectively.

To screen out the variables with high correlation, we performed Pearson's correlation analysis between all variables of five single-source remote sensing data sets and tea crown phenotype data. In addition, we selected 1–9 variables with high correlation as the input of single-source remote sensing data to establish the model (Figure 6). In Figure 6, ^*^ and ^**^ represent the significance levels of *P* < 0.05 and *P* < 0.01, respectively.

To evaluate the H of tea crown, L.Hmax, L.Hmean, L.H85th, and L.H95th variables of LiDAR were selected for modeling; P.Hmax, P.H65th, P.H75th, P.H85th, and P.H95th variables of TC were selected for modeling; RV, RContrast, GV, Gcontrast, and BV variables of RGB were selected for modeling; MEAN.B.450 and MEAN.R.660 variables of MS were selected for modeling; Tmax variable of TM was selected for modeling.

To evaluate the LAI of tea crown, L.Fgap, L.H35th, L.H45th, L.H55th, L.H65th, and L.H75th variables of LiDAR were selected for modeling; P.Fgap, P.H35th, P.H45th, and P.H55th variables of TC were selected for modeling; RV, Rcontrast, GV, Gcontrast, BV, and Bcontrast variables of RGB were selected for modeling; MEAN.R.660, PVI, RECI, and RENDVI variables of MS were selected for modeling; Tmax and Tmin variables of TM were selected for modeling.

To evaluate the W of tea crown, L.Fgap, L.H35th, L.H45th, L.H55th, L.H65th, and L.H75th variables of LiDAR were selected for modeling; P.Fgap, P.H15th, P.H25th, P.H35th, and P.H45th variables of TC were selected for modeling; RM, RASM, GM, GCorrelation, BM, and BASM variables of RGB were selected for modeling; SAVI, MNLI, GNDVI, and RENDVI variables of MS were selected for modeling; Tmax and Tmin variables of TM were selected for modeling.

To evaluate the LCC of tea crown, L.Fgap, L.H35th, L.H45th, L.H55th, L.H65th, and L.H75th variables of LiDAR were selected for modeling; P.Fgap, P.H15th, P.H 25th, P.H35th, and P.H45th variables of TC were selected for modeling; RM, RASM, RCorrelation, GM, GASM, GCorrelation, BM, BASM, and BCorrelation variables of RGB were selected for modeling; MEAN.RE.720, MEAN. NIR. 750, EVI, RDVI, and MNLI variables of MS were selected for modeling; Tmax and Tmin variables of TM were selected for modeling.

To evaluate the LNC of tea crown, L.Fgap, L.H35th, L.H45th, L.H55th, L.H65th, and L.H75th variables of LiDAR were selected for modeling; P.Fgap, P.H25th, P.H35th, and P.H45th variables of TC were selected for modeling; RM, RASM, RCorrelation, GM GASM, GCorrelation, BM, BASM, and BCorrelation variables of RGB were selected for modeling; MEAN.NIR. 750, MEAN. 840, EVI, SAVI, RDVI, MNLI, and GNDVI variables of MS were selected for modeling; Tmax and Tmin variables of TM were selected for modeling.

#### Performance of single-source UAV data on tea plant phenotyping

**Figure 7:**
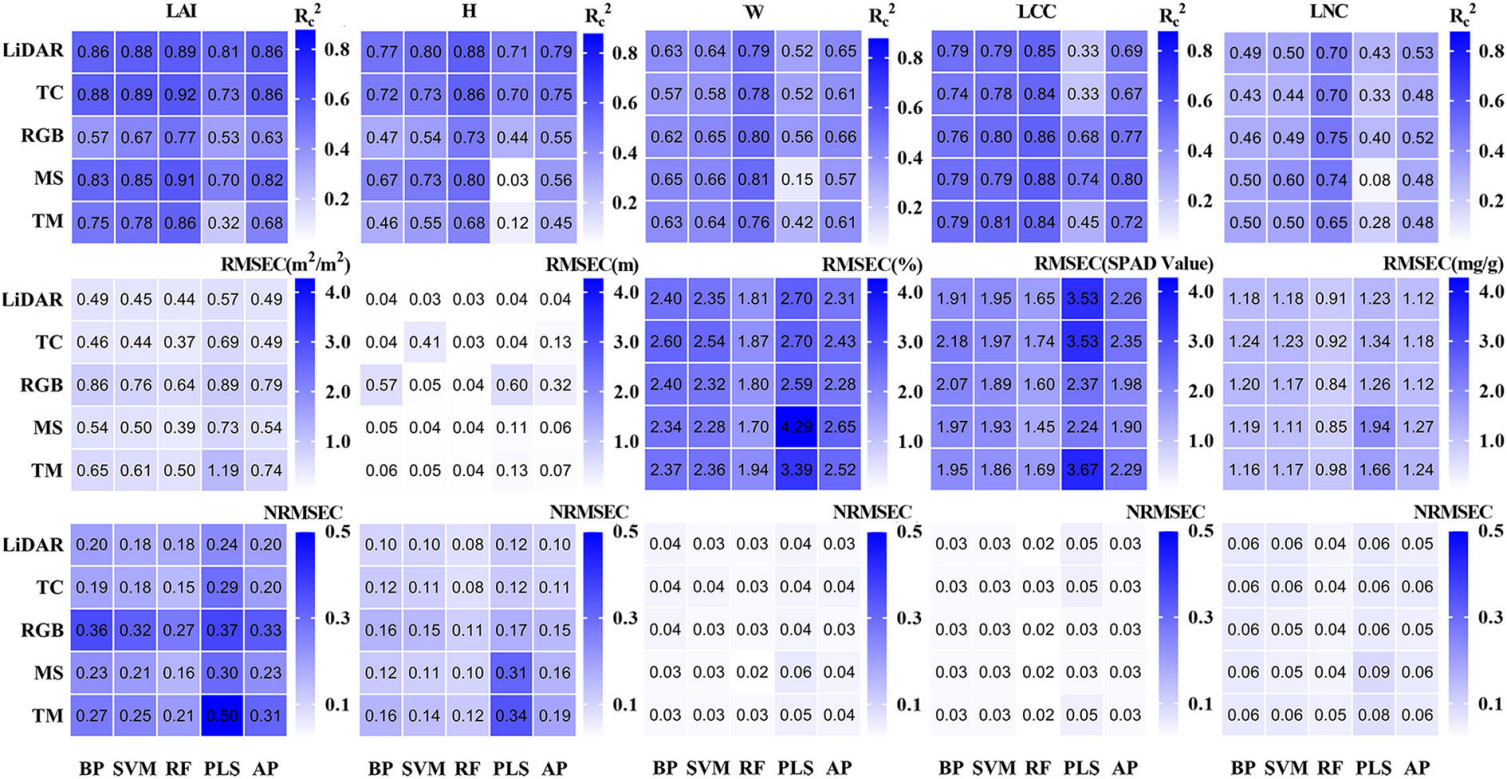
Result of the training set.

**Figure 8:**
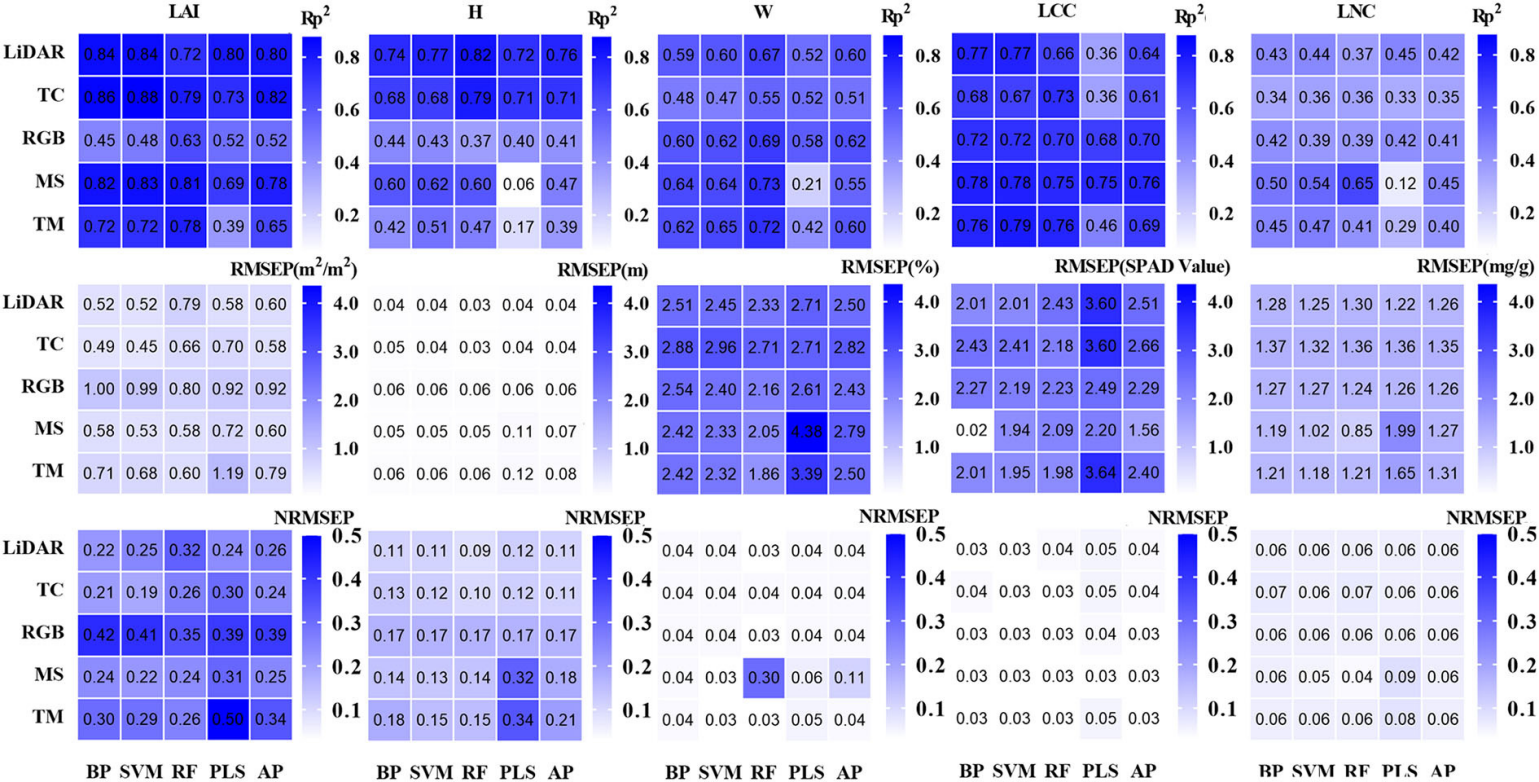
Result of the test set.

After selecting the appropriate single-source data, BP, SVM, RF, and PLS of machine learning methods were used to model the single-source remote sensing data and tea crown phenotype data. The results showed that the evaluation of crown phenotype by data from various sensors was significantly different (Figures 7, 8).

For the estimation of H, the model established by LiDAR and TC data has higher accuracy, and the AP value in the model established by LiDAR data is higher. The data from MS, RGB, and TM are not suitable for estimating tea plant height. The RF model established by LiDAR data has the best evaluation effect (Rp^2^ = 0.82, RMSEP = 0.031, and NRMSEP = 0.089).

For the estimation of LAI, the model established by TC, LiDAR, and MS data has high accuracy. In the model established by TC data, the AP value is the largest, so its stability is the highest. The SVM model established by TC data has the highest accuracy (Rp^2^ = 0.84, RMSEP = 0.45, and NRMSEP = 0.19).

For the estimation of W, the model established by RGB and TM data has high accuracy. In comparison, the AP value in the model established from RGB data is higher, so its stability is higher. The RF model established from TM data has the best evaluation effect (Rp^2^ = 0.72, RMSEP = 1.9, and NRMSEP = 0.03).

For the estimation of LCC, the performance and stability of the model established by MS data are the best. The BP model established by MS data has the highest accuracy (Rp^2^ = 0.78, RMSEP = 1.9, and NRMSEP = 0.029).

For the estimation of LNC, the AP value and accuracy of the model established by each data are low. In comparison, the RF model established by MS data has the highest accuracy (Rp^2^ = 0.65, RMSEP = 0.85, and NRMSEP = 0.04).

In conclusion, LiDAR and TC data are better in evaluating H and LAI of tea crowns. MS data are better in evaluating LAI, LCC, and LNC of tea crowns. RGB and TM data are better to evaluate the W of tea crowns.

### Contribution of multi-source remote sensing data to estimation of tea crown phenotypic

#### Screening of multi-source UAV remote sensing data

To evaluate the effect of multi-source remote sensing data on tea phenotype, we screened 2–7 variables with high correlation as the input of multi-source remote sensing data to establish model. To eliminate the influence of the number of variables on the comparison of single-source data and multi-source data, we keep the number of input variables unchanged. To evaluate the H of tea crown, L.Hmax, L.Hmean, P.H85th, and P.H95th variables of LiDAR + TC were selected for modeling. To evaluate the LAI of tea crown, L.Fgap, L.H45th, P.Fgap, and PVI variables of LiDAR + TC + MS were selected for modeling. To evaluate the W of tea crown, RM and Tmax variables of RGB+ TM were selected for modeling. To evaluate the LCC of tea crown, RM, MEAN.RE.720, MEAN.NIR. 750, EVI, and RDVI variables of RGB+ MS were selected for modeling. To evaluate the LNC of tea crown, L.Fgap, RM, BM, Tmax, SAVI, MNLI, and GNDVI variables of LiDAR + RGB + MS + TM were selected for modeling.

#### Performance of multi-source UAV data on tea plant phenotyping

**Figure 9:**
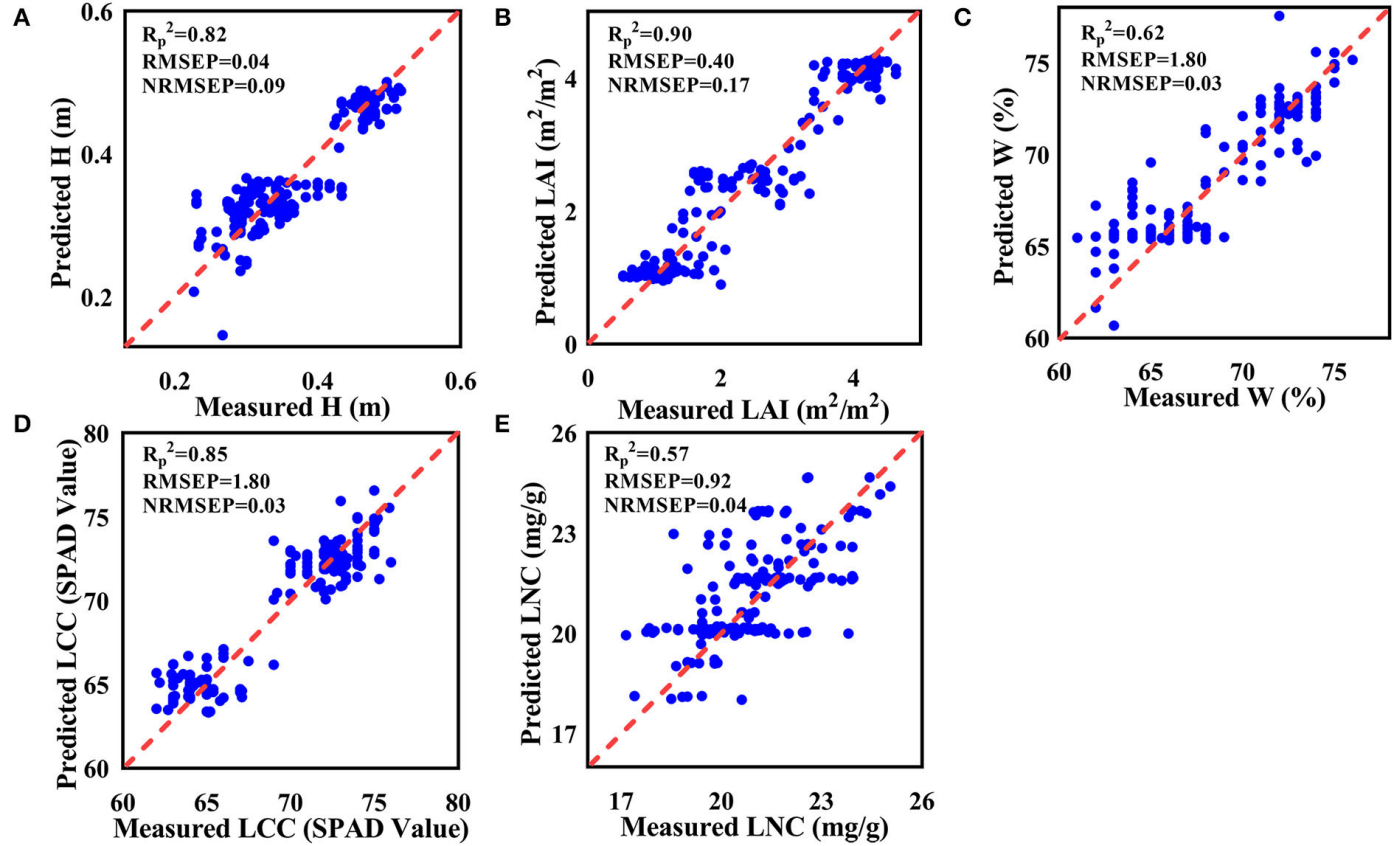
Scatter plot of predicted and actual values of the model. **(A)** H; **(B)** LAI; **(C)** W; **(D)** LCC; **(E)** LNC.

After selecting the appropriate multi-source remote sensing data, BP, SVM, RF, and PLS of machine learning methods were used to model the multi-source remote sensing data and tea crown phenotype data. The results show that the multi-source remote sensing data from multiple sensors have a good effect on the evaluation of tea crown phenotype (Table 5). For the estimation of H, the effect of SVM model is the best (Rc^2^ = 0.87, Rp^2^ = 0.82, RMSEC = 0.03, RMSEP = 0.04, NRMSEC = 0.078, and NRMSEP = 0.09); for the estimation of LAI, the effect of SVM model is the best (Rc^2^ = 0.91, Rp^2^ = 0.90, RMSEC = 0.39, RMSEP = 0.40, NRMSEC = 0.15, and NRMSEP = 0.17); for the estimation of W, the effect of SVM model is the best (Rc^2^ = 0.68, Rp^2^ = 0.62, RMSEC = 1.8, RMSEP = 1.8, NRMSEC = 0.03, and NRMSEP = 0.03); for the estimation of LCC, the effect of RF model is the best (Rc^2^ = 0.89, Rp^2^ = 0.85, RMSEC = 1.4, RMSEP = 1.8, NRMSEC = 0.02, and NRMSEP = 0.03); for the estimation of LNC, the effect of RF model is the best (Rc^2^ = 0.73, Rp^2^ = 0.57, RMSEC = 0.85, RMSEP = 0.92, NRMSEC = 0.04, and NRMSEP = 0.04). Figure 9 shows the scatter plot of the predicted value and actual value distribution of the model with the highest accuracy among the five phenotypic parameters.

**Table 5:** Phenotypic evaluation of tea plants based on multi-source remote sensing.

		**Training sets**			**Test sets**		
**Phenotype**	**Model**	**Rc^2^**	**RMSEC**	**NRMSEC**	**R^2^**	**RMSEP**	**NRMSEP**
H	BP	0.82	0.03	0.09	0.80	0.03	0.09
	SVM	0.87	0.03	0.08	0.82	0.04	0.09
	RF	0.90	0.02	0.07	0.81	0.04	0.09
	PLS	0.78	0.04	0.10	0.77	0.04	0.10
	AP	0.84	0.03	0.08	0.80	0.04	0.10
LAI	BP	0.9	0.4	0.16	0.88	0.46	0.19
	SVM	0.91	0.39	0.15	0.9	0.40	0.17
	RF	0.93	0.3	0.12	0.89	0.45	0.19
	PLS	0.84	0.5	0.21	0.84	0.51	0.22
	AP	0.89	0.39	0.16	0.85	0.49	0.22
W	BP	0.65	1.9	0.03	0.58	1.8	0.04
	SVM	0.68	1.8	0.03	0.62	1.8	0.03
	RF	0.78	1.8	0.03	0.49	1.9	0.04
	PLS	0.59	1.9	0.04	0.53	1.9	0.04
	AP	0.69	1.9	0.04	0.56	1.9	0.04
LCC	BP	0.78	2	0.03	0.75	1.9	0.03
	SVM	0.8	1.9	0.03	0.76	1.9	0.03
	RF	0.89	1.4	0.02	0.85	1.8	0.03
	PLS	0.75	2.1	0.03	0.74	2.2	0.03
	AP	0.81	1.85	0.03	0.79	2	0.03
LNC	BP	0.5	1.2	0.06	0.48	1.2	0.06
	SVM	0.52	1.2	0.06	0.46	1.2	0.06
	RF	0.73	0.85	0.04	0.57	0.92	0.04
	PLS	0.47	1.18	0.06	0.46	1.2	0.06
	AP	0.56	1.1	0.05	0.5	1.1	0.06

### Comparison of single-source and multi-source remote sensing data to evaluate the results of tea crowns phenotype

**Figure 10:**
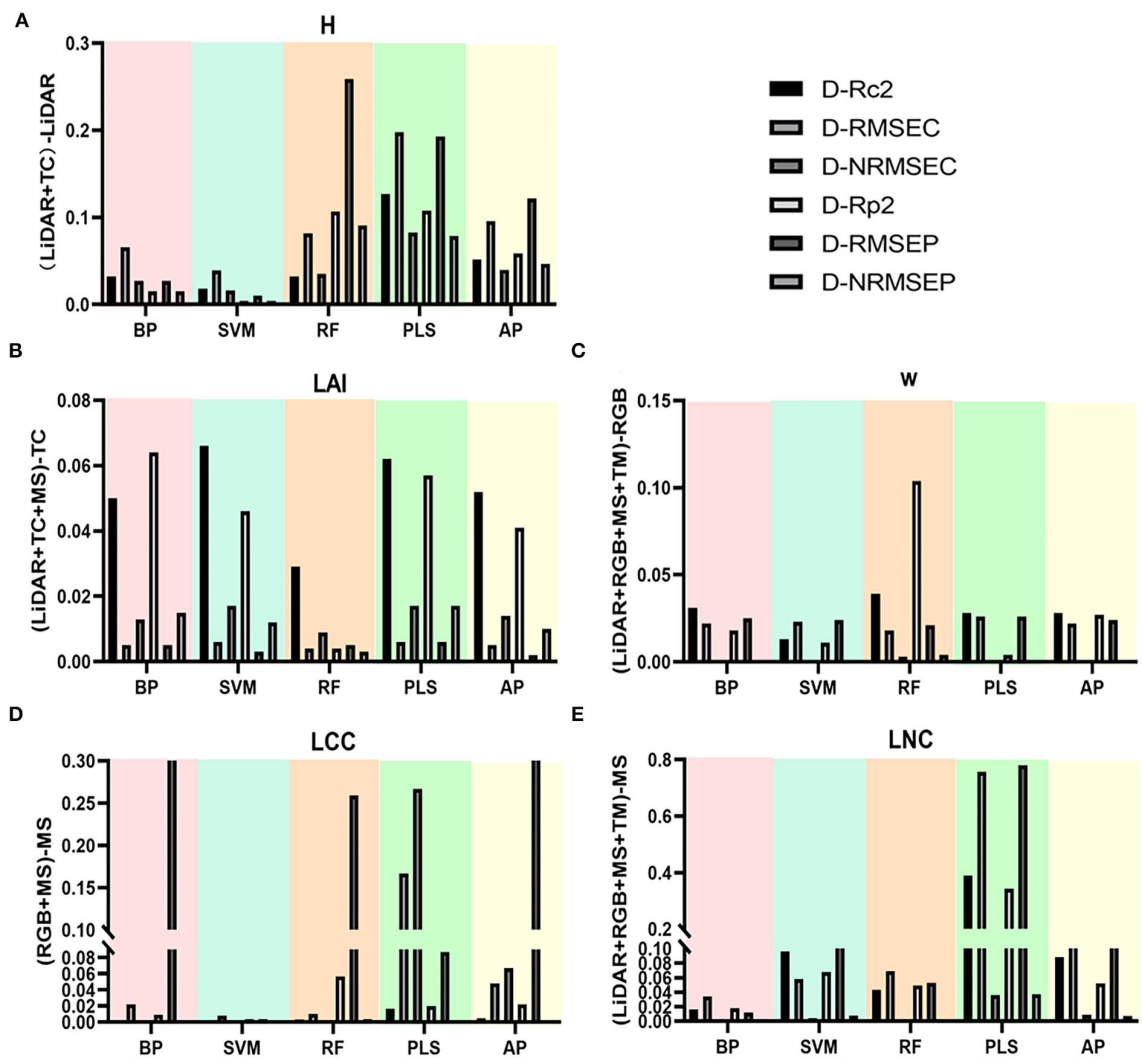
Evaluation results of single source and multi-source UAV data on tea crown phenotype. **(A)** H; **(B)** LAI; **(C)** W; **(D)** LCC; **(E)** LNC.

To more clearly and intuitively compare the evaluation results of multi-source remote sensing data and single-source remote sensing data on the phenotypic parameters of tea crowns, we calculated the difference between the evaluation results from the multi-source remote sensing data model and the evaluation results of the single-source remote sensing data model with the highest accuracy (Figure 10). To eliminate the influence of the number of variables, we keep the same number of variables input from multi-source data and single-source data. Table 6 shows the validation statistics of tea phenotypic parameters evaluated by single-source data set model with the highest accuracy and multi-source data set model. The results show that in evaluating the H of tea crowns, the accuracy of the model established by the fused LiDAR and TC data is greatly improved than that of LiDAR data, and the accuracy of the RF model is the highest. In evaluating the LAI of tea crowns, the accuracy of the model established by the fused LiDAR, TC, and MS data is improved than that of TC data; in evaluating the W of tea crowns, the accuracy of the model established by the fused RGB and TM data is greatly improved than that of RGB data, and the accuracy of the RF model is the highest. In evaluating the LCC of tea crowns, the accuracy of the model established by the fused MS and RGB data is greatly improved than that of the MS data. In evaluating the LNC of tea crowns, the accuracy of the model established by the fused LiDAR, MS, RGB, and TM data is significantly less than that of MS data, and the accuracy of the PLS model is the highest.

**Table 6:** Validation statistics of tea phenotypic parameters evaluated by single-source data set model with the highest accuracy and multi-source data set model.

**Phenotypic parameters**	**Sensor type**	**No. of feature**	**Metrics**	**BP**	**SVM**	**RF**	**PLS**	**AP**
H	LiDAR	4	Rp^2^	0.74	0.77	0.81	0.72	0.76
			RMSEP	0.040	0.039	0.031	0.043	0.038
			NRMSEP	0.11	0.11	0.089	0.12	0.11
	LiDAR+TC	4	Rp^2^	0.80	0.82	0.81	0.77	0.80
			RMSEP	0.034	0.036	0.036	0.037	0.036
			NRMSEP	0.094	0.094	0.092	0.10	0.095
LAI	TC	4	Rp^2^	0.86	0.88	0.79	0.73	0.81
			RMSEP	0.49	0.45	0.66	0.70	0.58
			NRMSEP	0.21	0.19	0.26	0.30	0.24
	LiDAR+TC+MS	4	Rp^2^	0.88	0.9	0.89	0.84	0.85
			RMSEP	0.46	0.42	0.45	0.51	0.49
			NRMSEP	0.19	0.19	0.19	0.22	0.22
W	TM	2	Rp^2^	0.55	0.6	0.49	0.49	0.52
			RMSEP	2.4	2.3	3.4	3.4	2.5
			NRMSEP	0.035	0.034	0.030	0.049	0.037
	RGB+TM	2	Rp^2^	0.58	0.62	0.49	0.53	0.56
			RMSEP	1.8	1.8	1.9	1.9	1.9
			NRMSEP	0.04	0.03	0.04	0.04	0.04
LCC	MS	5	Rp^2^	0.78	0.77	0.75	0.75	0.76
			RMSEP	2.2	1.9	2.1	2.2	1.6
			NRMSEP	0.029	0.028	0.030	0.032	0.03
	RGB+MS	5	Rp^2^	0.75	0.78	0.85	0.74	0.79
			RMSEP	1.9	1.9	1.8	2.2	2
			NRMSEP	0.03	0.03	0.03	0.03	0.03
LNC	MS	7	Rp^2^	0.50	0.54	0.65	0.12	0.45
			RMSEP	1.2	1.02	0.85	2.0	1.3
			NRMSEP	0.057	0.049	0.040	0.095	0.060
	LiDAR+RGB+MS+TM	7	Rp^2^	0.48	0.46	0.57	0.46	0.5
			RMSEP	1.2	1.2	0.92	1.2	1.1
			NRMSEP	0.06	0.06	0.04	0.06	0.06

## Discussion

### To select suitable single-source remote sensing data set to evaluate the phenotypic parameters of tea crowns

The results of this study verify that UAV remote sensing data sets from different sources are suitable for specific tea phenotypic parameters. LiDAR and TC sensors are dominant in monitoring H and LAI. The data obtained by the two sensors can establish a three-dimensional point cloud model to restore the crown structure of tea plants. Previously, researchers used LiDAR sensors to establish 3D point cloud models to monitor the forest canopy structure (Schneider et al., 2019). Some researchers also used oblique camera to establish 3D point cloud models to monitor the height and leaf area index of corn (Ying et al., 2020). These results are in accordance with our findings. The canopy structure of plants may be monitored using LiDAR and a TC camera. It is because LiDAR is an active sensor that the modeling accuracy of LiDAR data is higher than that of the TC data. The principle of LiDAR data acquisition is to transmit laser signals, which are reflected by tea plants and collected by the receiving system, so the penetration is better. However, we advocate utilizing TC cameras to monitor canopy structure for certain tea plants with low canopy structure. On the one hand, the TC camera can provide high-precision RGB data and 3D point cloud data; on the other hand, the TC camera is far less expensive than LiDAR.

RGB and MS sensors are dominant in monitoring LCC and LNC indexes of tea plants. Previous studies used multispectral data to estimate the nitrogen concentration of winter wheat (Tao et al., 2020). Here, we evaluated the accuracy of textural characteristics and spectral information in assessing the LCC of tea crowns, unlike aforementioned investigation. Our approach transforms in a model with a greater level of accuracy than one based on texture characteristics. We can utilize hyperspectral sensors for intensive analysis if we want to better monitor the LCC of the tea canopy, and hyperspectral sensors can collect more spectral information. Zhu et al. (2021) utilized hyperspectral data to determine the chlorophyll content of maize leaves.

The TM sensor has great potential in monitoring the water content of tea crown leaves. This was mainly because the thermal sensor could obtain the canopy temperature of crops, and there was a certain relationship between the temperature information and leaf water content (Luz and Crowley, 2010). In recent years, thermal sensors were more and more widely used in monitoring crop leaf water content (Maimaitijiang et al., 2020), such as researchers used thermal sensors to monitor changes in wheat moisture content and achieved good results (Abdelhakim et al., 2021). However, in this study, the accuracy of leaf water content prediction model is low, which may be due to the complexity and uncontrollability of field environment affecting the acquisition of temperature information. Therefore, if we can accurately obtain the changing trend of field environmental factors, such as wind speed, temperature, and humidity, it will help us to improve the accuracy of the model, which needs further research in the later stage.

### To select suitable multi-source remote sensing data set to evaluate the phenotypic parameters of tea crowns

In this research, the accuracy of the model established by the combined LiDAR and TC data is much greater than that of the model established by single-source data while evaluating H of tea crown. This may be due to the strong fault tolerance of multi-source data, which reduces the impact of environmental factors on specific types of data, improves spatial resolution, and enriches remote sensing image information; the accuracy of the model established by the fused LiDAR, TC, and MS data is small improved than that of the model established by single-source data while evaluating the LAI of tea crowns. However, previous researchers used multi-source remote sensing data to evaluate LAI of maize, which greatly improved the accuracy of the model (Liu et al., 2021). We analyze the reasons for the difference in accuracy between the tea plants and maize. On the one hand, because tea plants have the characteristics of high canopy density, especially in mature tea gardens, the measuring instrument is difficult to reach the center to measure the LAI. Therefore, there are errors in the measurement, which will affect the accuracy of the model. On the other hand, the evaluation of maize LAI is based on the fusion of RGB, MS, and TM data, while the evaluation of tea leaf area index is based on the fusion of LiDAR, TC, and MS data. Different data types may lead to different improvement of model accuracy. The accuracy of the model established by the fused LiDAR, MS, RGB, and TM data is much higher than that of the model established by single-source data while evaluating the W of tea crowns. Previously, researchers used the fusion of RGB texture features and vegetation index to evaluate the water content of rice, and the research results were consistent with our research results (Wan et al., 2020). However, different from our research method, we have more data types and larger amount of data to evaluate the water content of tea leaves, so the improvement of model accuracy is also greater. The accuracy of the model established by multi-source remote sensing data is improved than that of the model established by single-source remote sensing data while evaluating the LCC of tea crowns. Previously, multi-source remote sensing data were used to evaluate corn LCC and also proved that the accuracy of multi-source remote sensing data model is higher than that of single-source model in evaluating LCC (Zhu et al., 2021). The accuracy of the model established by MS data is improved than that of the model established by LiDAR + RGB + MS + TM data while evaluating the LNC of tea crowns. This may be because the LNC of tea plant only has a strong response to spectral information, but has a weak response to thermal information and texture information. At present, there are few literature on the evaluation of crop nitrogen content by multi-source remote sensing data.

### Effects of different machine learning algorithms on phenotypic evaluation of tea crown

While examining tea phenotypes, the accuracy of SVM, RF, and BP models is distinct by using a single data set. Among them, the SVM model has the highest accuracy in evaluating LAI, the RF model has the highest accuracy in evaluating H, W, and LNC, and the BP model has the highest accuracy in evaluating LCC. However, the stability of the BP model is low, and the accuracy decreases in the evaluation of LAI and W. As consistent with previous studies, using BP algorithm to establish corn leaf area index and leaf water content model, the number of samples is too small, resulting in low model accuracy. Therefore, BP neural network is suitable for large sample modeling. For small sample modeling, the stability is poor, and the parameters need to be adjusted constantly (Zhu et al., 2021). The accuracy of the PLS model is the lowest and in evaluating LCC and W of tea canopy, Rp^2^ <0.3. This is consistent with previous studies. SVM, BP, and PLS algorithms were used to build the prediction model of nitrogen, tea polyphenols, and amino acid content in tea leaves, of which the prediction model established by PLS algorithm had the lowest accuracy (Luo et al., 2021). This may be because the principle of PLS algorithm is combined with principal component analysis (PCA) to reduce the dimension of data. Although this will improve the running speed of the model, it will also lose some data information, resulting in low accuracy of the model (Wold et al., 2001). When applied to an evaluation of the tea phenotype using multi-source data, the RF and SVM modeling algorithms provide more accurate results. Among them, the RF algorithm is the most effective one for establishing LCC and LNC content prediction models of tea crowns. This is due to the fact that RF is able to balance the faults and errors of different types of data sets and is simple to parallelize (Yuan et al., 2017). SVM algorithm is better to establish LAI, H, and W content prediction models of tea crowns, and this is because the SVM network structure is more complex and has strong generalization and prediction ability (Yuan et al., 2017). The accuracy of BP model based on multi-source remote sensing data is higher than that of BP model based on single-source remote sensing data. This may be due to the increase in the number of samples, which increases the fitting degree of the model and gives full play to the advantages of BP algorithm.

In our research, we found that RF and SVM models have stable performance and high accuracy. Our results are consistent with other researchers using machine learning method to establish phenotypic models of rice and maize (Cen et al., 2019; Lin et al., 2021; Wang et al., 2021). We prefer to use RF algorithm, because RF algorithm has simpler network structure and faster running speed.

## Conclusion

In this study, the UAV is equipped with MS, TM, RGB, LiDAR, and TC sensors to monitor the tea height, leaf area index, leaf water content, leaf chlorophyll, and nitrogen concentration of the tea plantations in the three growth stages and obtain the structure information, spectral information, texture information, and temperature information of the tea plants. Remote sensing data were utilized to model with BP, SVM, RF, and PLS of machine learning algorithms, and the performance of single-source and multi-source remote sensing data sets to evaluate the crown phenotype of tea plants was studied. The main conclusions are as follows: On the one hand, using multi-source data sets to evaluate H, LAI, W, and LCC can greatly improve the accuracy and robustness of the model. For the evaluation of H, LiDAR + TC data sets are recommended for analysis, and SVM model provides the best estimation (Rp^2^ = 0.82 and RMSEP = 0.078). For the evaluation of LAI, LiDAR + TC + MS data sets are recommended, and SVM model provides the best estimation (Rp^2^ = 0.90 and RMSEP = 0.40). For the evaluation of W, RGB + TM data sets are recommended, and SVM model provides the best estimation (Rp^2^ = 0.62 and RMSEP = 1.80). For the evaluation of LCC, MS +RGB data set is recommended for analysis, and RF model provides the best estimation (Rp^2^ = 0.87 and RMSEP = 1.80). On the other hand, using single-source data sets to evaluate LNC can greatly improve the accuracy and robustness of the model. For the evaluation of LNC, MS data set is recommended for analysis, and RF model provides the best estimation (Rp^2^ = 0.65 and RMSEP = 0.85).

## Data availability statement

The original contributions presented in the study are included in the article/supplementary material, further inquiries can be directed to the corresponding author.

## Author contributions

HL carried out the experiment, collected and organized data, processed the multi-source remote sensing data, and wrote the manuscript. YM provided the modification of the article's picture. ZD, YW, and KF proposed the hypothesis for this work, designed the experiment, helped organize the manuscript structure, and directed the study. YS participated in the design of the experiment and directed the study. All authors contributed to the article and approved the submitted version.

## Funding

This research was funded by the Significant Application Projects of Agriculture Technology Innovation in Shandong Province (SD2019ZZ010), the Technology System of Modern Agricultural Industry in Shandong Province (SDAIT-19-01), the Special Foundation for Distinguished Taishan Scholar of Shangdong Province (ts201712057), the Livelihood Project of Qingdao City (19-6-1-64-nsh), and the Project of Agricultural Science and Technology Fund in Shandong Province (2019LY002, 2019YQ010, and 2019TSLH0802).

## Conflict of interest

The authors declare that the research was conducted in the absence of any commercial or financial relationships that could be construed as a potential conflict of interest.

## Publisher's note

All claims expressed in this article are solely those of the authors and do not necessarily represent those of their affiliated organizations, or those of the publisher, the editors and the reviewers. Any product that may be evaluated in this article, or claim that may be made by its manufacturer, is not guaranteed or endorsed by the publisher.
